# Modeling long-term human activeness using recurrent neural networks for biometric data

**DOI:** 10.1186/s12911-017-0453-1

**Published:** 2017-05-18

**Authors:** Zae Myung Kim, Hyungrai Oh, Han-Gyu Kim, Chae-Gyun Lim, Kyo-Joong Oh, Ho-Jin Choi

**Affiliations:** 1 School of Computing, KAIST, 291 Daehak-ro, Yuseong-gu, Daejeon, 34141 South Korea; 2 Samsung Seoul R&D Campus, Samsung Electronics, 33 Seongchon-gil, Seocho-gu, Seoul, 06765 South Korea

**Keywords:** Heart rate, Calorie, Footstep, Activeness prediction, Time series modeling, Recurrent neural network

## Abstract

**Background:**

With the invention of fitness trackers, it has been possible to continuously monitor a user’s biometric data such as heart rates, number of footsteps taken, and amount of calories burned. This paper names the time series of these three types of biometric data, the user’s “activeness”, and investigates the feasibility in modeling and predicting the long-term activeness of the user.

**Methods:**

The dataset used in this study consisted of several months of biometric time-series data gathered by seven users independently. Four recurrent neural network (RNN) architectures–as well as a deep neural network and a simple regression model–were proposed to investigate the performance on predicting the activeness of the user under various length-related hyper-parameter settings. In addition, the learned model was tested to predict the time period when the user’s activeness falls below a certain threshold.

**Results:**

A preliminary experimental result shows that each type of activeness data exhibited a short-term autocorrelation; and among the three types of data, the consumed calories and the number of footsteps were positively correlated, while the heart rate data showed almost no correlation with neither of them. It is probably due to this characteristic of the dataset that although the RNN models produced the best results on modeling the user’s activeness, the difference was marginal; and other baseline models, especially the linear regression model, performed quite admirably as well. Further experimental results show that it is feasible to predict a user’s future activeness with precision, for example, a trained RNN model could predict–with the precision of 84%–when the user would be less active within the next hour given the latest 15 min of his activeness data.

**Conclusions:**

This paper defines and investigates the notion of a user’s “activeness”, and shows that forecasting the long-term activeness of the user is indeed possible. Such information can be utilized by a health-related application to proactively recommend suitable events or services to the user.

## Introduction

With advances in technology and ever-busy schedules, people tend to lack physical activity, and have increased level of stress. They are hence at a greater risk of suffering from the so-called “modern diseases” such as cardiovascular disease, diabetes, metabolic disorders, and stroke [1]. Attaining a healthy lifestyle, which incorporates a balanced diet and a plenty of exercise, is considered to be key in preventing such diseases.

Recently, many health-care-related devices and services have emerged to aid users in monitoring and improving their physical wellness (Section Devices and services for wellness improvement). With wearable devices, such as fitness trackers, it has been possible to continuously observe the biometric data produced by a user, and notify the user when he/she has been physically inactive for a period of time. Many services also provide users with general tips on a healthy lifestyle, and motivation to be physically more active during the day, for example, by letting them know how many steps remain to reach the weekly average, or by offering them virtual “badges” to commemorate their physical achievements which can be boasted over a social media platform.

While some of these approaches have been considered to be effective by their users [2], this paper suggests that their usefulness can be further improved if a long-term predictive model of the user’s “activeness” is incorporated into the health-care services. For example, an application can project the user’s activeness for some period of time in the future, and inform him/her of the remaining days before the weight loss goal is (or not) reached. In addition, it may take a more proactive measure, depending on the user’s context, and preemptively recommend possible exercises that he/she could perform when the activeness is predicted to be below a threshold (Section Finding time windows with low activeness).

Many research efforts have been made to accurately model and predict users’ heart rates [3–6] and energy expenditures [7–9], often as a mean to recognize their simple activities (e.g. walk, run, lying down, etc.) [5] or to identify any medically significant event such as heart failure [4, 6, 10].

As the task of activity recognition or detection of heart failure often involves classifying a relatively short span of time, most existing works utilize machine learning algorithms such as feed-forward neural networks (FFNNs), support vector machines (SVMs), and random forests (RFs) that are known to be effective in learning short-term temporal dependencies among time-series data. Furthermore, these works often employ wearable sensors that are specifically designed for a certain type of biometric data, and focus on building an accurate model for the type of data and the task at hand.

In this paper, we slightly shift the perspective, and aim to investigate the feasibility of modeling a user’s long-term activeness which could, to some extent, represent his/her lifestyle pattern. Currently, our notion of activeness for a given period of time is tracked as a tuple of heart rate, consumed calories, and the number of footsteps taken by the user.

Instead of utilizing separate wearable sensors for each type of data, a fitness tracker is used to continuously record the three types of biometric data of the user for several months (Section Data set construction). We experiment with recurrent neural network (RNN) architectures which are considered to be well suited for learning long-term dependencies among temporal data. While there are many studies of RNN architectures being applied to various sequential modeling tasks (Section Recurrent neural networks), few works exist in the domain of wellness modeling. Therefore, this paper explores how the performance of activeness prediction is varied by changing (1) a set of length-related hyper-parameters of the training process, and (2) the RNN architectures.

The rest of the paper is organized as follows. Section Background explores the background for this study, while Section Methods illustrates the proposed approach. In Section Results and discussion, we describe and analyze the gathered time-series dataset, and present the experimental results. Finally, Section Conclusion concludes the paper with some directions for future works.

## Background

This section briefly introduces some of the commercial devices and services that are proposed to measure and improve a user’s “wellness” (Section Devices and services for wellness improvement), along with some academic researches that aim to model biometric data for various tasks (Section Modeling biometric data). Moreover, existing works that involve time-series modeling using RNNs are presented in Section Recurrent neural networks.

### Devices and services for wellness improvement

According to the Centers for Disease Control and Prevention, USA, 70.7% of American adults over the age of 20 are overweight, and 37.9% of the same group are obese as of 2013–2014 [11]. As a need for a “fitness revolution” is greater than ever before, fitness devices and services are flooding the marketplace.

Since 2006, the footwear company Nike has introduced the “Nike+ Sports Kit” that records the distance and paces of a walk or run, and transmits the data to the user’s smart device. A series of all-around activity trackers have been independently manufactured by Fitbit and Jawbone. These fitness trackers measure the number of steps taken and log the heart rates of the wearer. Based on these measurements (and other biometric information), the consumed calories and the traveled distance are calculated. This study utilized Jawbone’s “UP3” model and Fitbit’s “Charge HR” model to continuously record users’ heart rate, footsteps, and calories.

Several fitness centers and health-care providers have devised wellness “scores” or “indices” that aim to quantify the physical fitness of an individual. For example, Life Time Fitness proposes the “myHealthScore” [12] that is determined by six indicators: blood pressure, triglycerides, total cholesterol to high-density lipoprotein ratio, glucose, body fat, and tobacco use.

Dacadoo introduces the “Health Score” [13] which ranges from 1 to 1000, and is calculated from biometric values (gender, age, weight, waist circumference, blood pressure, etc.), emotional values (acquired from self-assessment questionnaires), and lifestyle values (exercise, nutrition, steps, sleep, etc.). Linking with the aforementioned fitness trackers, the Health Score is continuously updated throughout the day as the user performs activities such as walking, running, sleeping, etc.

The “Wellness Score” [14] offered by 8 Weeks to Wellness ranges from 1 to 100, and is calculated using various biomarkers including: body mass index, posture number, core strength and flexibility, body fat percentage, and heart rate.

While these measures claim to represent an individual’s state of wellness or health, how the corresponding factors are combined to produce a single value is not known publicly. Furthermore, there is not yet a general consensus even among doctors and medical researchers about what constitutes wellness and how they should be defined and measured. For example, several key dimensions can exist to define wellness–physical, psychological or emotional, social, intellectual, spiritual, occupational, environmental, cultural, economic, and climate–and for each dimension, different researchers may view certain factors more important than other factors, and thus propose different scoring functions [15].

In addition, the holistic perspective of calculating a single wellness score is not fully grounded on medical examination; after all, the involved factors vary in both characteristics and units.

For these reasons, we specify that this study targets to model a person’s physical “activeness”, which is kept as a series of tuples of heart rate, consumed calories, and the number of footsteps, and avoid using the more general term, “wellness”.

### Modeling biometric data

While the term “biometric data” in the context of security, generally refers to measurable physical characteristics that help authenticating an individual (e.g. fingerprint, retina, vein, etc.), this study refers to its more general meaning–the measurable biological quantities of an individual that, unlike the former kind, may change over time. This paper targets three types of biometric data–heart rate, burned calories (energy expenditures), and the number of footsteps–that reflect how physically active a person is for a given period of time.

The task of modeling human heart rates and energy expenditures (EE) has been widely studied across many disciplines such as sports science, medicine, electrical engineering, and computer science. Keytel et al. [7] developed a prediction equation for EE from the heart rate by monitoring 115 regularly exercising individuals aged 18 to 45 years old. The participants performed exercises on a treadmill, and their heart rate and respiratory exchange ratio data were collected. A mixed model analysis identified gender, heart rate, weight, maximal oxygen uptake, and age as important factors in estimating EE.

Cheng et al. [3] proposed a non-linear state-space control system that modeled the heart rate of a person walking on a treadmill, and later utilized the model to build a computer-controlled treadmill system for regulating the heart rate during exercises.

Sumida et al. [5] introduced an approach that predicted the heart rate of a walking user, utilizing an accelerometer and GPS data obtained from the user’s smartphone. The authors used the raw data from the smartphone to calculate the oxygen uptake, which was then fed as a form of input data to an artificial feed-forward neural network (FFNN).

Similarly, Pande et al. [8] estimated EE for ambulatory activities using accelerometer and barometer sensors in a smartphone. Their model, which also used an artificial FFNN, outperformed calorimetry equations and EE values obtained from commercial fitness trackers.

Bouarfa et al. [9] targeted a slightly more general setting of estimating EE under “free-living” conditions using a single ear-worn accelerometer. A regression analysis was used to predict EE values, while linear discriminant and nearest neighbor classifiers were employed to classify a window of accelerometer values into one of ten activities such as lying down, standing, computer work, vacuuming, etc. The regression model correlated well with the medical gold standard, the doubly labeled water test.

In addition to the general task of modeling heart rate and EE, some works specifically focus on medical problems such as heart failure detection. For example, Austin et al. [4] compared the classification performance of several machine learning algorithms such as logistic regression, bagging, RF, and SVM, when applied to classifying patients with heart failure (HF) into one of two mutually exclusive subtypes: HF with preserved ejection fraction and HF with reduced ejection fraction. In their study, however, a set of detailed clinical data of patients was utilized as opposed to the time-series data.

More relevantly, Zheng et al. [10] proposed a multi-channel deep convolutional neural network (MC-DCNN) for a time-series classification. Their model was applied to a set of electrocardiograph data which had been recorded from 15 patients suffering from severe congestive heart failure. The task was to classify a 2D time-series input into one of four types of heartbeats. In their experiments, the proposed MC-DCNN performed better than the nearest neighbor approaches and FFNNs.

Our work is similar to the above studies in that we aim to model a person’s heart rates, burned calories (EE), and number of footsteps. However, while the above works generally consider time-series data with lengths from a few seconds to a dozen minutes, this work aims to model a long-term temporal pattern by considering much longer periods of temporal data, ranging from a dozen minutes to days.

### Recurrent neural networks

**Fig. 1:**
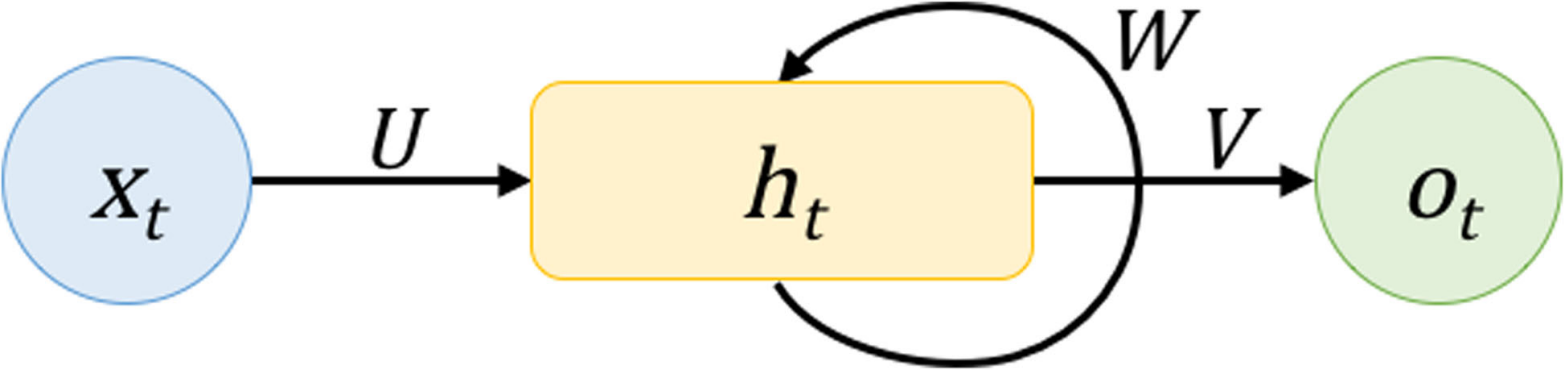
General structure of vanilla RNNs

Recurrent neural networks (RNNs) represent a class of artificial neural networks where some connections between nodes form a directed cycle (Fig. 1).

Fundamentally, RNNs carry out the same task for every element of an input sequence (*x*_*t*_), producing an output (*o*_*t*_) that is both dependent on the current input and results from previous computations ($h_{t_{1}}$). For example, in the case of a vanilla RNN depicted in Fig. 1, the hidden state (*h*_*t*_) at time *t* is computed by:

1 $$  h_{t}=f_{h}\left(Ux_{t}+Wh_{t-1}\right)  $$

where *U* and *W* represent the learned weight matrices that are multiplied to vectors *x*_*t*_ and *h*_*t*−1_ respectively; the non-linear function *f*_*h*_(·) is usually a hyperbolic tangent function (tanh) or a rectified linear unit (ReLU).

The output sequence (*o*_*t*_) is then calculated by:

2 $$  o_{t}=f_{o}(Vh_{t})  $$

where *V* and *f*_*o*_(·) denotes the learned connection weight matrix for *h*_*t*_ and the output unit activation function.

In essence, these two non-linear equations describe a dynamic system where the future behavior of a real-world system is captured deterministically by learning from the series of past observations. Such learned information is captured in the state of the dynamic system which, in the case of RNNs, corresponds to the set of hidden unit activations (*h*_*t*_). Therefore, the modeling power (or complexity) of a dynamic system is determined by its state space as well as its input and output spaces. Again, in the context of RNNs, the order of state space corresponds to the number of hidden units.

An RNN’s recursive loop can be “unfolded” over time, which converts the network into a feed-forward neural network. This means that a standard backpropagation algorithm can be applied to the unfolded RNN for training. However, this also means that the classical problem of vanishing or exploding gradients [16] may be present in the training process. To avoid this problem, researchers have sophisticatedly formulated the internal structure of the hidden state. Notable examples include the long short-term memory (LSTM) unit, first conceived by Hochreiter and Schmidhuber [17], and the gated recurrent unit (GRU) by Cho et al. [18]. Unlike the simple structure of the hidden state of the vanilla RNNs, these units incorporate a delicate gating mechanism that effectively enforces constant error flow and overcomes the saturation of gradients. A detailed investigation of the LSTM family is conducted by Greff et al. [19].

The recurrent behavior of RNNs has made them an effective solution for various tasks involving sequential data modeling: stock markets [20], energy consumption [21], genetic expression [22], speech [23], and language modeling [24]. In the medical domain, RNNs are often used to model physiological signals such as electrocardiograms [25–27]. Recently, Lipton et al. [6] applied LSTM cells to the task of multi-label classification of multivariate clinical time-series data. While the task was to predict the probability distribution of 128 labels (e.g. diabetes, asthma, scoliosis, neoplasm, etc.), the authors improved the performance of the model via auxiliary output training which utilized the remaining 301 diagnostic labels.

## Methods

To the best of our knowledge, modeling a person’s long-term “activeness” using RNN architectures has not been studied previously. This work utilizes GRU cells to model the biometric data, exploring different layouts of networks and parameter settings.

In this work, we are interested in predicting the activeness of a person based solely on his/her previous data. As mentioned in Section Recurrent neural networks, such task of temporal sequence modeling can be effectively conducted using RNN architectures. This study explores four RNN architectures described in Section Network architectures.

### Task settings

**Fig. 2:**
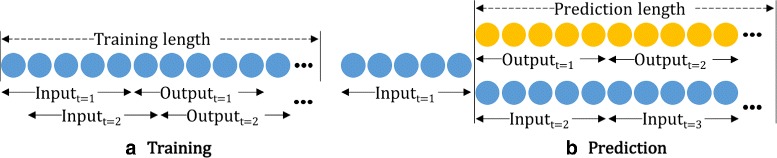
Length-related hyper-parameters for **a** training, and **b** testing
- **Input length** corresponds to the number of time steps that the network takes as an input. When modeling a single type of biometric data, a memory cell of LSTM or GRU receives one-dimensional vector at a time step *t*, and updates its cell state using the current input vector *x*_*t*_ and the previous cell state *c*_*t*−1_. Therefore, the input length *n* determines how many time steps are processed internally by the memory cell before producing an output vector *o*_*t*_ at *t*=*n*. In a typical case of language modeling, the input length is often set to the average (or maximum) sentence length. However, in our scenario of modeling activeness, it is not so apparent as to how long should the time steps be for each user. Hence, this study explores the variations on this parameter.
- **Output length** refers to the length of the time-series data that the model is required to predict for a given input data.
- **Prediction length** represents the total length of the time-series data that we want to predict.

Before looking at the architectures, it is necessary to identify the hyper-parameters for training and evaluating an RNN model for activeness prediction as they directly affect the performance of the model. Figure 2 illustrates the length-related hyper-parameters for (a) training, and (b) testing a network (prediction).

- **Training length** specifies the total length of the time-series data that are used to train the model.

We note that other network-based hyper-parameters such as regularization methods, and choices in loss and activation functions can also affect the network’s performance. This study primarily focuses on exploring the impact of aforementioned length parameters on the activeness prediction.

### Network architectures

**Fig. 3:**
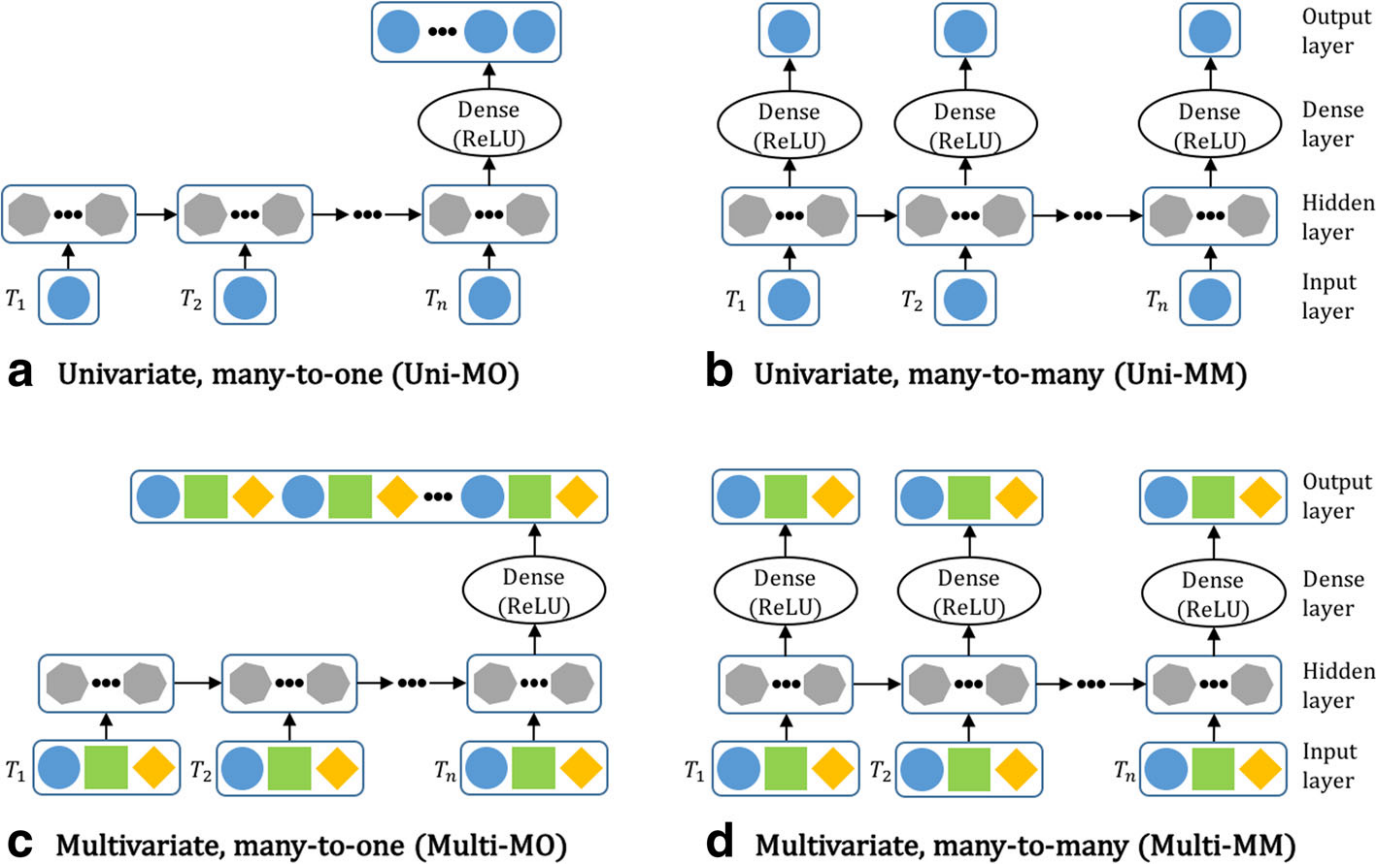
The four types of recurrent neural network architectures: **a** univariate, many-to-one (Uni-MO), **b** univariate, many-to-many (Uni-MM), **c** multivariate, many-to-one (Multi-MO), and **d** multivariate, many-to-many (Multi-MM)

As there are infinite number of ways in structuring a neural network model, building an effective network architecture requires much practice and patience. In this study, we experiment with the following four RNN architectures–univariate many-to-one (Uni-MO), univariate many-to-many (Uni-MM), multivariate many-to-one (Multi-MO), and multivariate many-to-many (Multi-MM)–depicted in Fig. 3.

Architectures (a) and (c) are each formulated in the many-to-one fashion where the output is computed only at the last time step of an input data. Notice that these two structures offer more flexibility in choosing the output length than many-to-many approach, (b) and (d), in the sense that the output length can be different to the input length. In many-to-many approach, an output vector is computed at each time step, and is in the same dimension as the input vector.

A univariate architecture models each type of biometric data separately, while a multivariate architecture considers the three types of data together in the same model. Therefore, for each user, three univariate models are trained as we have three types of activeness data, while just one model is built for the multivariate architecture.

## Results and discussion

### Data set construction

Our experiments utilize three types of biometric time-series data: heart rate, number of steps walked, and amount of calories burned. Seven graduate students between the ages of 23 to 33 years old participated their biometric data. It is noted that as the participants were all graduate students, the gathered data could be biased towards the group as less active as opposed to a more active group of “athletes” or “outdoor service employees”.

However, a simple survey was conducted and revealed that the participants’ lifestyle patterns were quite different to one another. For example, three participants described themselves as regularly exercising, while differing in the type and duration of the workouts. Also, the participants’ bedtimes and wake-up times were not congruent as well. An extreme case was a participant who operated on a three-day cycle, where he stays up for two days and sleeps for the next entire day.

A Jawbone’s UP3 fitness tracker was worn by each participant, and used to gather the three types of the biometric data. The device is equipped with a tri-axis accelerometer that detects physical movements, and a bio-impedance sensor that measures heart rate, respiration, and galvanic skin response. While the exact internal logic for the tracker is not known publicly, we believe that the consumed calories are calculated via its own energy expenditure equation that considers the wearer’s age, body mass index, number of footsteps, and heart rate.

Recently, some criticisms have been made on the accuracy of the estimated calories [28], pointing out that various fitness trackers compute different amount of burned calories when worn by the same user simultaneously. Nevertheless, our experimental objective does not necessitate an impeccable accuracy in computing the exact value of energy expenditures as the main task is to learn the long-term trend.

As the heart rate data in BPM were recorded at an irregular interval (ranging from a few seconds to a few minutes), a linear interpolation was conducted to prepare the data in one-minute-intervals. For the burned calories and number of footsteps, when an activity of an arbitrary duration was performed, the total sum of each type of data was recorded for that activity. Therefore, for each time stamp in the duration of the activity, we assigned the mean value, and later augmented the values to fit them in one-minute-intervals.

After the interpolation or the augmentation, the values underwent a log transformation as they are heavily positively skewed. Min-max normalization is then applied to the data in order to fit them in 0 to 1 range. The minimum and maximum values for each type of data were selected by consulting relevant medical documents.

The statistics of the gathered data are presented in Table 1. For convenient comparison among the users, we prepared the time-series data to begin at the same time stamp. The number of samples represents the number of minutes in the recorded duration.

**Table 1:** Statistics of the gathered time-series data for the seven users

User	Start time	End time	Duration (months)	Number of samples
AK	2015-10-12 00:00:00	2016-05-04 14:21:00	6.85	296,062
HJ		2015-11-29 02:32:00	1.60	69,273
JM		2015-11-19 19:05:00	1.29	55,866
KJ		2016-05-02 23:57:00	6.80	293,758
YJ		2016-05-03 18:00:00	6.83	294,841
YS		2016-01-14 10:11:00	3.15	135,972
ZM		2016-04-15 06:53:00	6.21	268,254

### Data set analysis

Before conducting the main experiments, it would be beneficial to inspect the data set for any noticeable characteristic or pattern. First, we check if the three types of activeness data are correlated to each other by computing the Pearson product-moment correlation coefficients. The coefficient ranges between -1 to +1 inclusive, and represents the strength of linear dependence between two variables *X* and *Y*, where 1 is total positive linear correlation; 0 is no correlation; and -1 is total negative correlation. The values are computed using the raw time-series data before the log and min-max normalization. The results are presented in Table 2.

**Table 2:** Pearson product-moment correlation coefficients among the three types of activeness data

	Calorie	Footstep	Heart rate
Calorie	1	0.88506139	0.03684186
Footstep	0.88506139	1	0.04260965
Heart Rate	0.03684186	0.04260965	1

The calories and footsteps are positively correlated as these data were generated at the same time when an activity was sensed by the tracker. However, it is interesting to note that there seems to be no correlation between the heart rate and the other two types of data. One possible explanation would be the user’s activities, in general, were mostly of mild intensity, and hence did not result in high beats per minute. It is also possible that a user would perform an activity of high intensity, such as weight training, that did not require many footsteps to take.

**Fig. 4:**
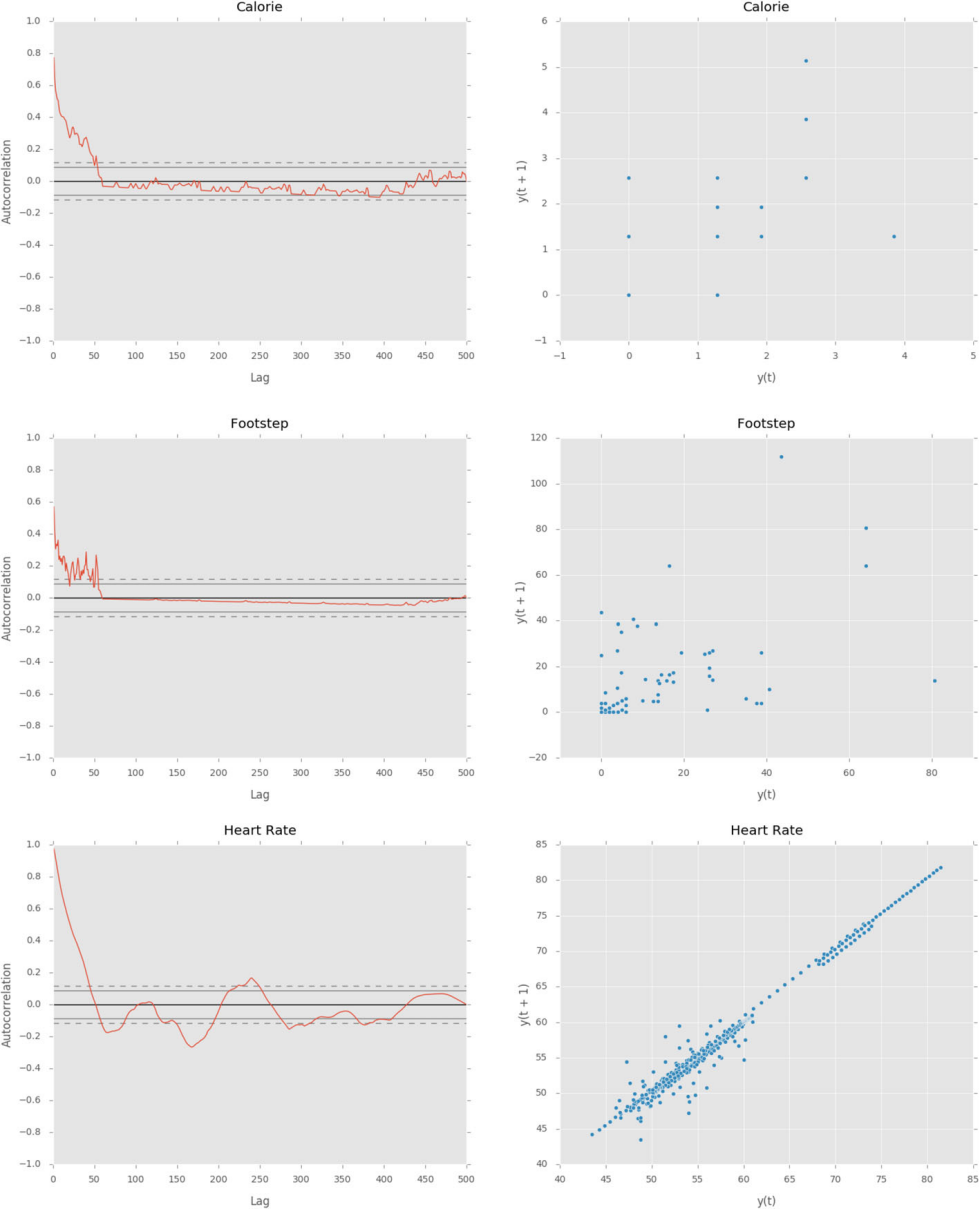
Autocorrelation graphs (*left*) and lag plots (*right*) for each type of data

Another standard time-series analysis is checking the correlation between values of a single variable, i.e., autocorrelation. Positive autocorrelation reflects the “persistence” of a system, where the system tends to remain in the same state from a time step to the next. In Fig. 4, the graphs on the left-hand side illustrate the autocorrelation among each type of data of a user, while the plots on the right-hand side depict the current activeness values, *y*_*t*_, against the next values, *y*_*t*+1_. The dashed and solid horizontal lines in the autocorrelation graphs show 99% and 95% confidence bands respectively.

The autocorrelation graphs illustrate that the footsteps and calories tend to correlate with itself only at the beginning, i.e., the first 50 min. This hints that the user, at that time, did not perform an activity that is longer than 50 min. This is more apparent in the footstep graph where the autocorrelation drops to almost zero after 50 min. The autocorrelation graph of heart rate depicts slightly different pattern–there is a one major peak at 250 min. It is often understood in signal processing domain that such peak in the autocorrelation graph corresponds to the cycle of the signal. In this context, however, as we have not observed such recurring pattern for a long time, it would be a little hasty to assume such pattern. It would seem that the user perhaps had encountered an event that excited his mental state which, in turn, increased the heart rate.

The lag plots on the right-hand side illustrate if the time-series is random or not. A random series would not show any identifiable pattern, while a non-random one would exhibit a coherent pattern, such as the linear pattern shown for the heart rate data. This linear pattern suggests that fitting a linear model to the heart rate data would be more effective than to calorie and footstep data as the two types of data show rather sporadic patterns.

### Choosing an RNN architecture

While separately conducting all experiments on each of the four RNN architectures would be ideal, due to time and resource constraints, we select the best performing one to be the specimen for the subsequent experiments. We individually train each RNN architecture using the first one month of the time-series data, and generate the data for the next week; the input and output length is kept constant at 15 min for convenient comparison.

In addition to these length parameters, GRUs are selected to be the memory cells for our RNN architectures as they converged faster than LSTM cells while preserving the accuracy. Each architecture is trained to minimize the mean squared error (MSE) using a recently proposed optimization method called Adam [29]. The training process of a model is terminated when no improvement is made on a randomly chosen set of unseen time-series data. We also note that a dropout rate of 0.2 is used in every layer for regularization, and rectified linear unit (ReLU) is chosen to be the non-linear activation function for the fully-connected (dense) layers as it is known to be robust to the vanishing gradient problem.

Each architecture is trained using a single user’s activeness data. As there are seven users in total, we have seven separate models for each of the two multivariate architectures, Multi-MO and Multi-MM. For each of the two univariate architectures (Uni-MO and Uni-MM), we have 21 models (3∗7) as there are three types of activeness data to be modeled separately. In addition, because we are varying the number of hidden units, every model is trained for all variations. On a single GeForce GTX TITAN X graphic card, training one model under the current length parameter setting took up to 90 min.

The predicted results of the seven users are evaluated under symmetric mean absolute percentage error (SMAPE):

3 $$  SMAPE\left(y,\hat{y}\right)=\frac{100\%}{n}\sum\limits_{t=1}^{n}\frac{|\hat{y_{t}}-y_{t}|}{(|y_{t}|+|\hat{y_{t}}|)/2}  $$

SMAPE measures the proportion of prediction error relative to the magnitudes of both the predicted and correct values. Hence, the lower the SMAPE is, the more accurate the model is on modeling the data set. It can be viewed as a normalization of mean absolute error so that a direct comparison between the users is made possible.

**Fig. 5:**
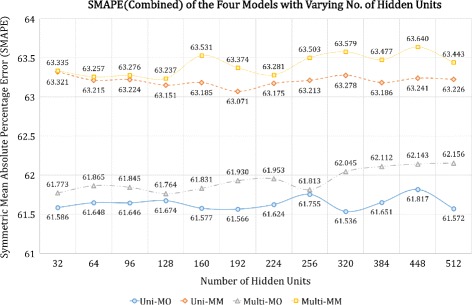
SMAPE for each RNN architecture with varying no. of hidden units

For each architecture, we take the mean of the seven users’ SMAPE for the three types of activeness data. In addition, for each user, we average the three types of SMAPE to compute the “combined” SMAPE. Figure 5 illustrates the combined SMAPE results for the four models.

We note that the result for each type of activeness data also produced graphs with the similar pattern shown in Fig. 5. However, we specify that while the SMAPE for heart rate ranged between 4 to 8%, that of calorie and footstep ranged between 82 to 93%.

We can make two main observations from the graphs:

1. The univariate architectures perform better than the multivariate ones.
2. The many-to-one architectures outperform the many-to-many ones.

In Fig. 5, the first observation reflects our previous finding that the heart rate data do not correlate highly with the calorie and footstep data; thus, feeding all three data to the model actually resulted in lower performance.

The second observation is more prominently shown in Fig. 5–it turns out that arranging the network in the many-to-one layout significantly improves the performance. This is perhaps due to the fact that all three data types autocorrelated well with their early subsequent values, and thus deferring the judgment until the last time step conveyed richer information than producing hidden activation pattern at every time step.

The increase in the number of hidden units did not result in better performance, if anything, slightly worse. We believe such phenomenon happens due to the overfitting of the model to the training data. A more fine-grained grid search for the range between 32 to 128 units revealed that 52 units produced the best result for the Uni-MO model. Therefore, for the subsequent experiments, a Uni-MO model with 52 hidden units were chosen to be the representative model for the RNNs.

### Effect of varying length parameters

Five experiments were conducted to evaluate the effect of the four length parameters on the performance of activeness prediction. We specify that when one length parameter was varied, the other three parameters were fixed as follows: training length=1month; input length=15mins; output length=15mins; and prediction length=1week. Table 3 summarizes the experiment setting.

**Table 3:** Five experiments on varying the length parameters [M=months, W=weeks, D=days]

Experiments	Input length (minutes)	Output length (minutes)	Training length	Prediction length
#1: Varying the input length alone	5/15/30/60/120	15	1M	1W
#2: Varying the output length alone	15	5/15/30/60/120	1M	1W
#3: Varying the input & output length alone	5/15/30/60/120		1M	1W
#4: Varying the training length alone	15		1W/2W/1M/3M/5M	1W
#5: Varying the prediction length alone	15		1M	1D/3D/1W/1M/3M

**Fig. 6:**
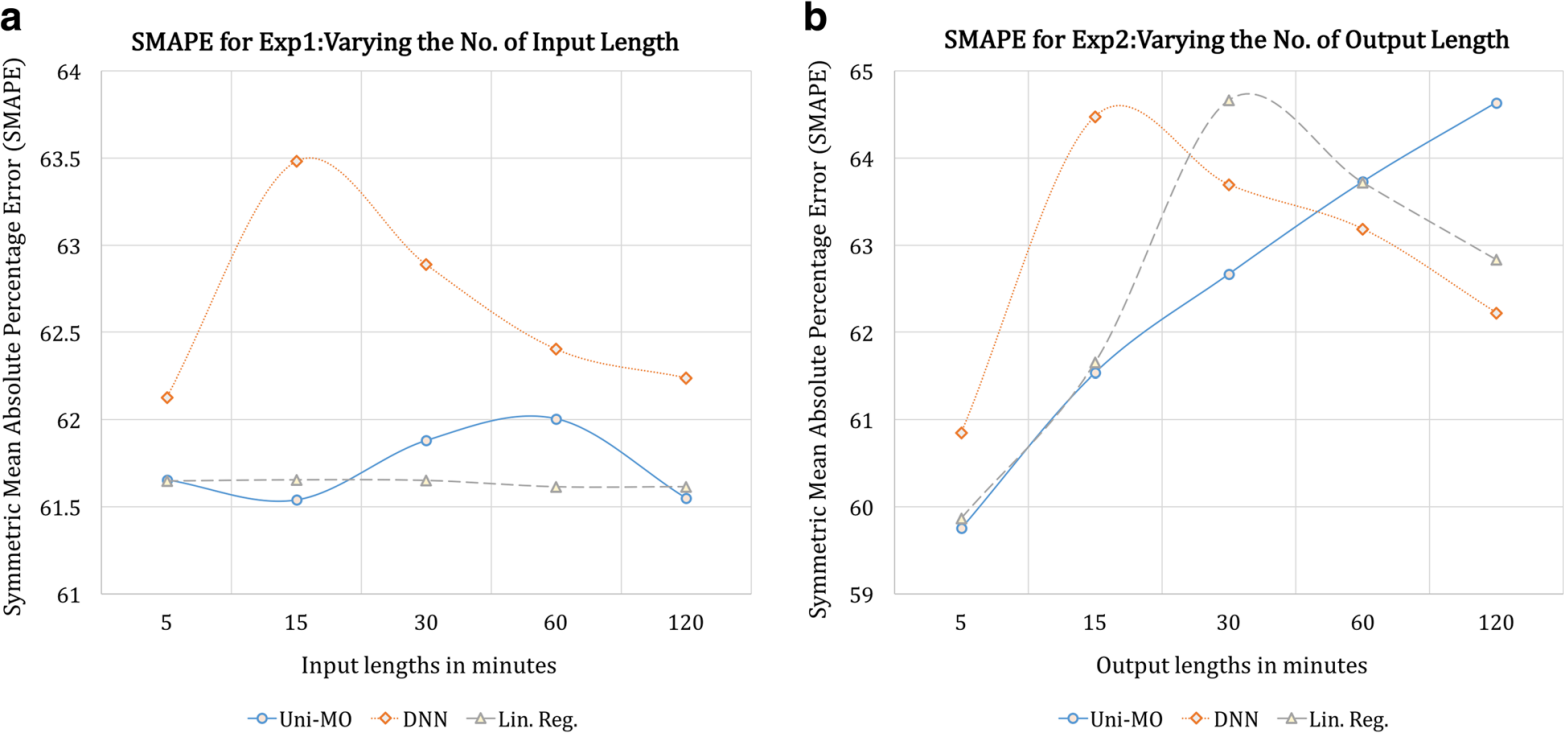
SMAPE of the three models for the first (**a**) and second (**b**) experiment

**Fig. 7:**
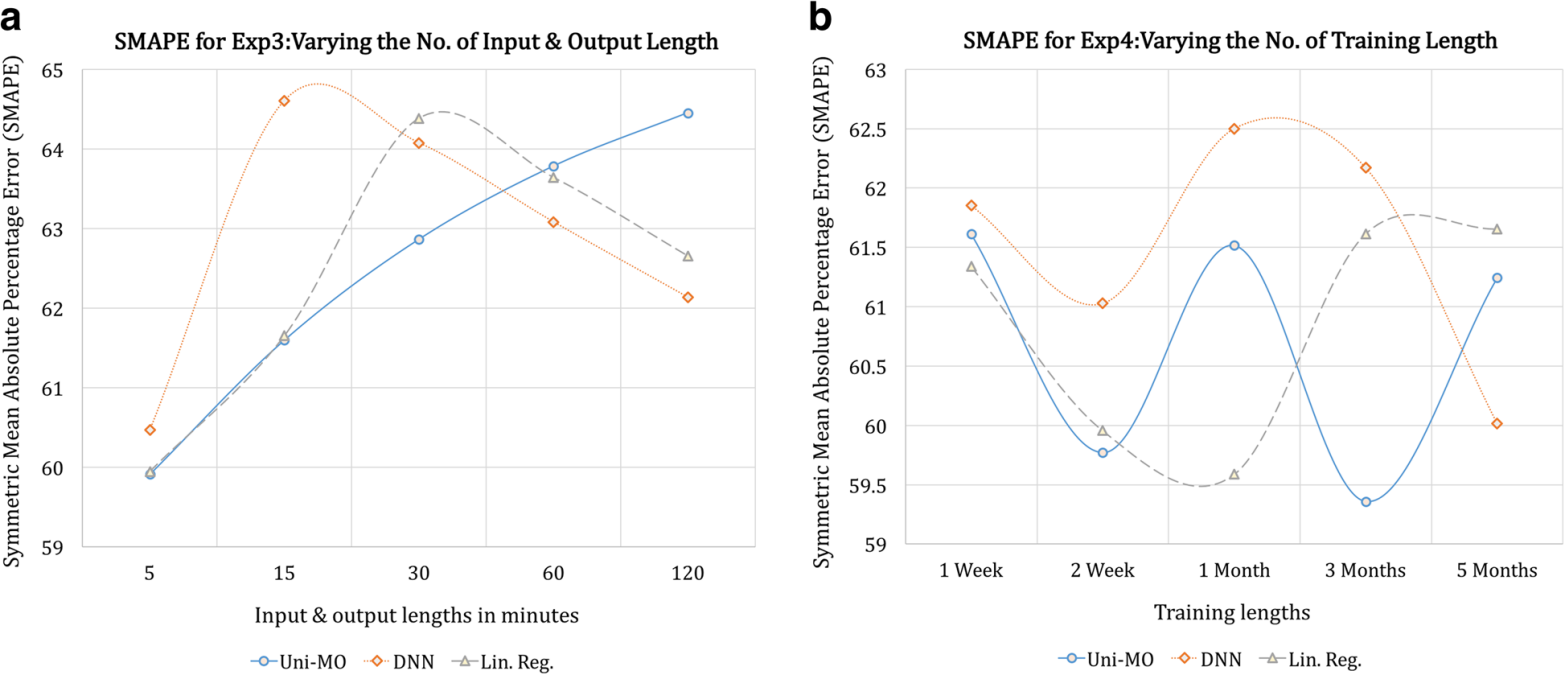
SMAPE of the three models for the third (**a**) and fourth (**b**) experiment

**Fig. 8:**
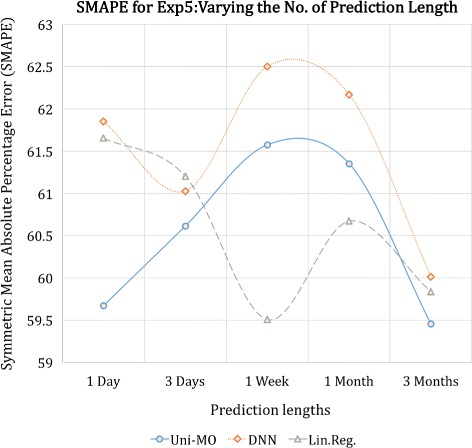
SMAPE of the three models for the fifth experiment

Uni-MO model with 52 hidden units were employed to conduct the experiments. We also utilized a deep neural network (DNN) with two fully connected layers (52 and 26 hidden units with ReLU as the activation function for both layers) and a linear regression model based on ordinary least squares method as baseline algorithms. Each experiment was conducted using the seven users’ data separately, and the their SMAPE values were averaged for comparison. Figures 6, 7, and 8 illustrate the results of the five experiments.

In Fig. 6-(a), we see that an increase in input length does not result in immediate performance gain. In fact, the top performance of DNN was achieved when the input length was set to 5 min; and RNN showed the smallest error when the length was set to 15 min. It appears that as the autocorrelation was strong only at the initial time steps, predicting the next 15 min was effectively done by just observing the first 5 to 10 min.

As for varying the output length in Fig. 6-(b), the performance was at its highest for all models when the output length was kept the shortest. The errors then increased for longer lengths.

In Fig. 7-(a), the result of the third experiment shows nearly the identical pattern to that of the second experiment, showing that even when the input and output lengths were varied together at the same time, the variation of the output length played the dominant role in terms of performance.

The result of the fourth experiment in Fig. 7-(b), varying the training length, shows a distinct pattern for each model. The lowest error was achieved by the RNN model when three months of training data were used. The regression model also showed good performance when the training length was set to 1 month.

Lastly, Fig. 8 illustrates the result of the fifth experiment–changing the prediction length. All three models reached their top performance when three months of unseen data were predicted. One possible explanation is that the test data up to the first month exhibited quite a different pattern to the training data, whereas the test data up to the first three months were a long enough duration that contained more regular patterns.

All in all, under the optimal hyper-parameter setting for each model, the RNN and linear regression model bested the DNN model, and the RNN model outperformed the regression model, albeit not by much. This illustrates a rather surprising finding that, to some extent, modeling a user’s activeness can be conducted quite effectively using a simple regression method, probably due to the fact that the data mainly exhibited short-term temporal dependencies.

In retrospect, setting the default training length to one month was rather an unfair experimental choice for the RNN model since the difference in errors between the RNN and regression model was particularly large when the training length was set to one month (Fig. 7-(b)). We believe that had the default training length been set to three months, the RNN model would have shown more noticeable superiority over the regression model. Similarly, in Fig. 8, we observe that the performance of the RNN model was at its lowest when the prediction length was set to the default length–one week. Nevertheless, the fact that the RNN model showed the best performance for all experiments suggests that it is a more powerful and stable modeling technique to use.

The limitation of the experiments lies in the small number of training samples for longer length parameters, i.e., we need to observe a user for longer period of time, and acquire sufficient number of samples to claim statistically more significant results. In addition, although we presented the averaged results for all seven users, every user had a distinct activeness pattern (Section Qualitative observations), and therefore would likely to produce different results when the individual is observed separately.

**Fig. 9:**
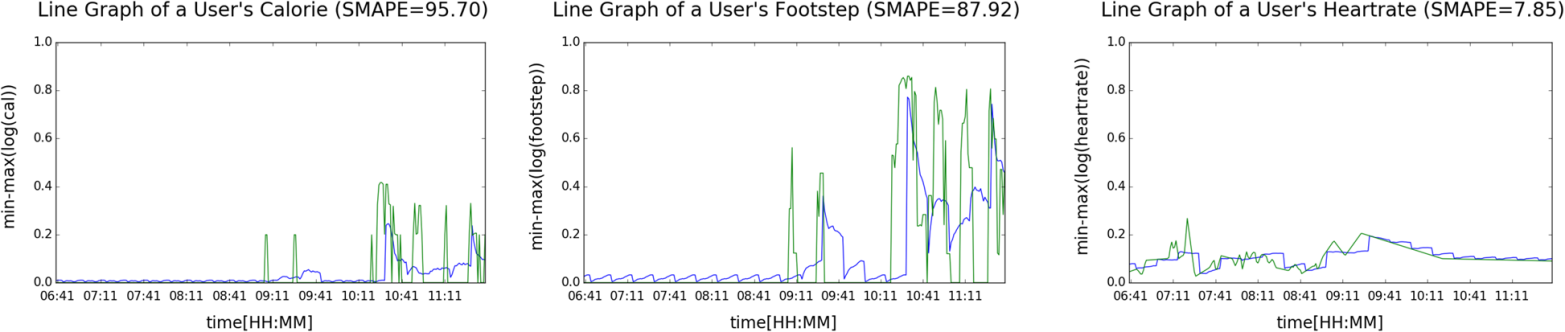
Predicting calorie, footstep, and heart rate of a user [output length = 15]

**Fig. 10:**
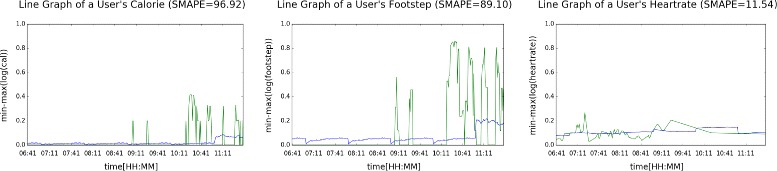
Predicting calorie, footstep, and heart rate of a user [output length = 60]

Figures 9 and 10 plot the predicted (blue) and real (green) values of activeness data of a user during one morning, where the former illustrates the results when output length was set to 15 min, and the latter, 60 min. We can see that predicting 60 min of unseen data given 15 min of input data was considerably a tougher task.

### Finding time windows with low activeness

From an application’s perspective, it would be acceptable to predict when a user will be inactive or less active. For those users who are predicted to be inactive in future moments, a health-care application may recommend some relevant exercises or alerts to the user depending on his/her context.

In this experiment, we aim to find the inactive time periods of a user utilizing our model for activeness prediction. We propose two ways in defining the time windows with low activeness:

- *Definition 1* A time window, whose length is equal to 15 min in our case, is marked as less active if more than 70% (10.5 min) of the window’s values are lower than the daily average value. Since there are three types of activeness, we would have three separate sets of time windows.
- *Definition 2* We take the intersection of the three sets of time windows defined in *Definition 1*, producing a single set of time windows. This means that a time window is marked as less active when all three type of activeness are below the corresponding daily average. This appears to be a more appropriate definition as a user might conduct exercises that require little walking or running, for example, performing a set of weight training exercises.

In order to find these time windows with low activeness, we experimented with the following two approaches:

- *Approach 1* Comparing the time windows predicted by the learned model against the true time windows of each type of activeness data.
- *Approach 2* Using all three (heart rate, footstep, calorie) models, devise a voting method such that a time window is taken to be less active if two of the three models predict it to be so.

The precision, recall, and f1-score results for the task are presented in Table 4.

**Table 4:** Precision, recall and f1-score results for finding the time windows with low activeness

Approach	Definition	Heart Rates			Calories			Footsteps		
		Pr.	Rc.	F1	Pr.	Rc.	F1	Pr.	Rc.	F1
Approach 1	Definition 1	.66	.66	.66	.78	.75	.75	.80	.77	.78
	Definition 2	.70	.60	.61	.72	.61	.62	.74	.63	.64
Approach 2	Definition 2	Precision=.84, recall=.65, f1-score=.66								

It seems that following the *Definition 2* presented a tougher task than the *Definition 1*. It is also notable that the voting method, *Approach 2*, improved the classification performance.

### Qualitative observations

**Fig. 11:**
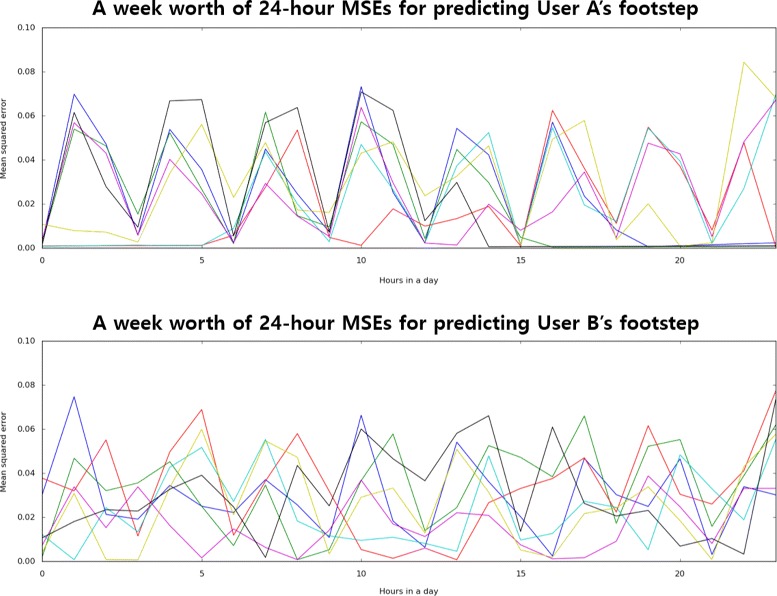
Predictability of two users, A and B, with regular (user A) and irregular (user B) weekly lifestyle. The colored line represents each of the seven days

Looking through the prediction results obtained by the different users, we observed that a user with a regular lifestyle was indeed easier to predict than a user with an erratic lifestyle (Fig. 11). For example, the graphs below illustrate the mean squared error (MSE) for predicting two users’ footsteps over seven days. The seven colored line represents the seven days in the week.

The MSE for predicting the user A’s footstep is decreased significantly at certain times in a day, implying that at those times, the user had been very predictable. However, the graph of the user B consistently shows high MSE, implying that his/her lifestyle had not been very consistent during the week.

## Conclusion

In this work, we explored the feasibility of modeling a user’s activeness using biometric data retrieved from fitness trackers. We proposed four RNN architectures, and later selected one (Uni-MO) to further investigate the performance under various length parameter settings. We observed that although the top results were achieved by the RNN model, a simple linear regression model also performed admirably, which reflected the short-term temporal dependencies among the time-series data. Through the additional experiment on predicting the time windows with low activeness, we saw that forecasting when a user would be less active was indeed feasible with good precision.

For future works, we plan to gather activeness data of participants with more diverse lifestyle, and investigate if a cluster of people, for instance, “morning people”, can be formed; and if a set of rules that describe the general pattern of an individual’s activeness data can be extracted [30]. In addition, we are currently developing a health-care application that aims to increase a user’s activeness through proactively recommending (and learning) activities that the user likes to perform. By observing the user in the long run, we hope to see if such application has a prominent effect on the user’s activeness.
